# lncRNA ACTA2-AS1 predicts malignancy and poor prognosis of triple-negative breast cancer and regulates tumor progression via modulating miR-532-5p

**DOI:** 10.1186/s12860-022-00432-7

**Published:** 2022-07-27

**Authors:** Yi Peng, Xiaoxi Huang, Hongmei Wang

**Affiliations:** Department of Breast Surgery, Fujian Provincial Maternity and Children’s Hospital, 18 Dao Shan Road, Gulou District, Fuzhou, 350001 China

**Keywords:** Triple-negative breast cancer, lncRNA ACTA2-AS1, miR-532-5p, Prognosis, Development

## Abstract

**Background:**

Dysregulation of ACTA2-AS1 and miR-532-5p and their functions in various cancers have been widely reported. Their potential of serving as biomarkers in triple-negative breast cancer (TNBC) remains unknown. This study aimed to evaluate the function of ACTA2-AS1 and miR-532-5p and their potential of serving as biomarkers in TNBC.

**Results:**

The TNBC tissues were collected from 119 patients, where the reduced level of ACTA2-AS1 and increased level of miR-532-5p were observed by PCR and showed a significantly negative correlation (*P* <  0.001). Both ACTA2-AS1 and miR-532-5p were closely associated with the malignant development and poor prognosis of TNBC patients. Moreover, in TNBC cell, overexpressing ACTA2-AS1 was found to suppress cell proliferation and metastasis, which was reversed by the upregulation of miR-532-5p.

**Conclusions:**

ACTA2-AS1 and miR-532-5p could act as biomarkers of TNBC predicting the progression and prognosis of patients. ACTA2-AS1 served as a tumor suppressor of TNBC which was mediated by miR-532-5p.

**Supplementary Information:**

The online version contains supplementary material available at 10.1186/s12860-022-00432-7.

## Introduction

The incidence of breast cancer in women is frequent in the past decades, although increasing efforts have been made in improving the diagnosis and therapy of breast cancer. In China, the incidence of breast cancer is increasing year by year [1]. As the early symptoms of breast cancer are insignificant, patients are always diagnosed at an advanced stage with distant metastasis, systemic multiorgan lesions, and even threatening patients’ lives [2–4]. The clinical prognosis of patients is closely associated with the pathological types, including Luminal A, Luminal B (HER2(+) and HER2 (−)), HER2-overexpressed, and triple-negative type [5]. Among these subtypes, triple-negative breast cancer (TNBC) is the most malignant subtype. Although the onset of TNBC only accounts for 15–20% of breast cancer patients, its 5-year survival rate is much lower than that of Luminal A, Luminal B (HER2(+) and HER2 (−)), and HER2-overexpressed breast cancer [6]. Due to the lack of specific biomarkers, the diagnosis of TNBC is mainly based on the pathological examination of needle biopsy, which is invasive and with low sensitivity. Additionally, TNBC patients are always diagnosed at an advanced stage, which limits the therapeutic strategies of patients, and the recurrence and metastasis are lately detected. Exploring novel indicators involved in the occurrence and metastasis of TNBC could benefit screening and monitoring TNBC progression and therefore ameliorate patients’ outcomes.

An increasing number of researches have been devoted to the long or short non-coding RNAs (lncRNAs and miRNAs) in human disease, especially to the dysregulated ncRNAs. A previous study identified a series of differently expressed ncRNAs in breast cancer from the Cancer Genome Atlas, including 55 lncRNAs, 88 miRNAs, and 1465 mRNAs, where lncRNA ACTA2-AS1 (lncRNA actin alpha 2, smooth muscle antisense RNA1) was found to be downregulated [7]. The function of ACTA2-AS1 has been illustrated in prior studies, such as colon adenocarcinoma, lung adenocarcinoma, and gastric cancer, with abnormal expression [8–10]. The upregulation of miR-532-5p in TNBC was leaked out in a previous expression profile of miRNAs in breast cancer at different onset stages [11], and its enhanced effect was also revealed in the cellular processes of breast cancer [12]. miR-532-5p was evidenced as the competing endogenous RNA (ceRNA) of ACTA2-AS1, by which ACTA2-AS1was able to play as a tumor promoter [13]. Whether ACTA2-AS1 was involved in the development of TNBC and whether it also regulated miR-532-5p during its function in TNBC remain unknown, which are of great significance in exploring novel biomarkers for TNBC.

This study concentrated on 119 TNBC patients, through evaluating the dysregulation of ACTA2-AS1 and miR-miR-532-5p, their clinical significance in TNBC development was disclosed. Moreover, with the employment of in vitro cell experiments, the roles of ACTA2-AS1 and miR-532-3p in the biological function of TNBC cells, including cell metastasis and proliferation were estimated, to provide new insight into the biomarker identification of TNBC.

## Results

### Expression of ACTA2-AS1 and miR-532-5p in TNBC and their correlation

**Fig. 1:**
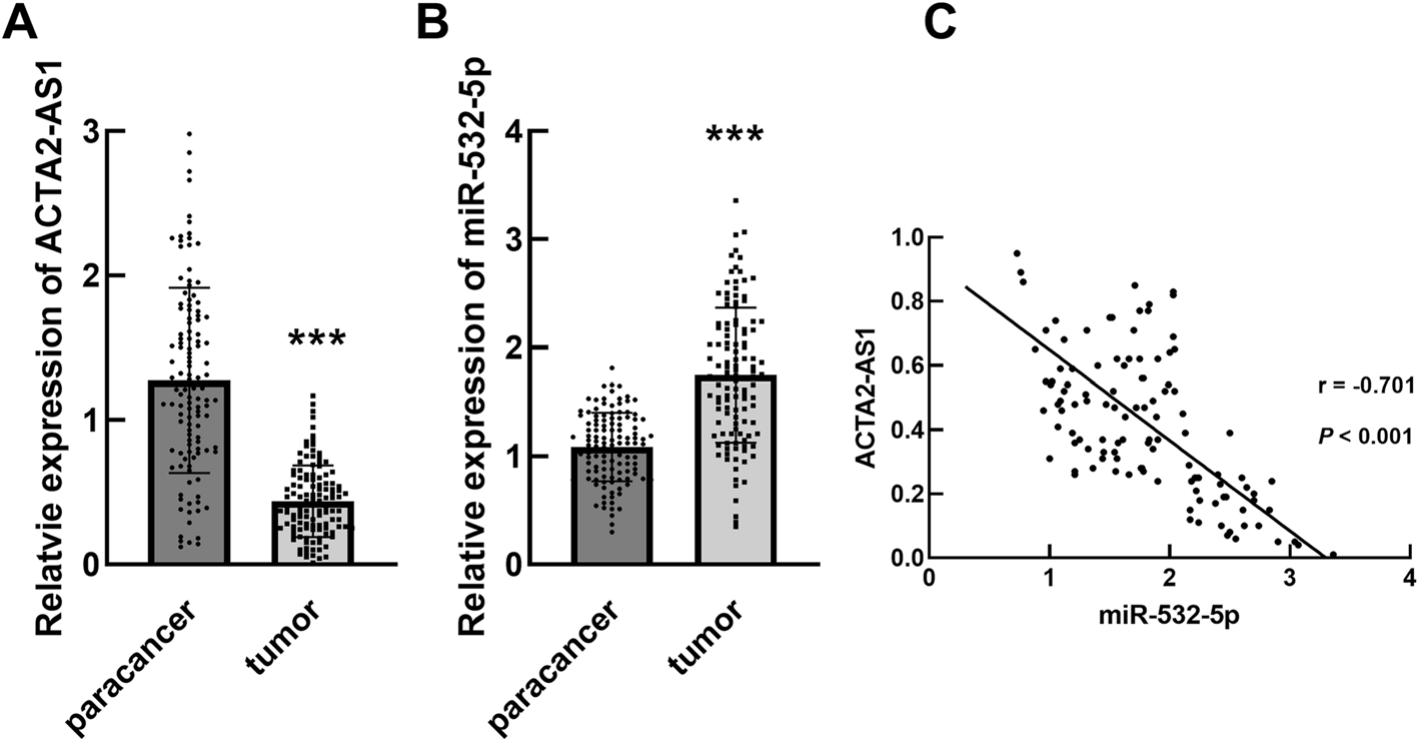
Expression of ACTA2-AS1 and miR-532-5p in TNBC tissues. **A**, **B** Significant downregulation of ACTA2-AS1 (**A**) and upregulation of miR-532-5p (**B**) were observed in TNBC tumor tissues. ^***^P < 0.001 compared with normal paracancerous tissues. **C** The expression of miR-532-5p was negatively correlated with the expression of ACTA2-AS1. *r* = − 0.701, *P* < 0.001

In tumor tissues, ACTA2-AS1 was found to significantly downregulate (Fig. 1A), while miR-532-5p was dramatically upregulated relative to the normal paracancerous tissues (Fig. 1B, *P* < 0.001). Meanwhile, ACTA2-AS1 was negatively correlated with miR-532-5p in tumor tissues (Fig. 1C, *r* = − 0.701, *P* <  0.001).

### Association of ACTA2-AS1 and miR-532-5p with patients’ clinicopathological features

Patients were grouped according to the mean of ACTA2-AS1 and miR-532-5p expression in TNBC tissues. It was found that ACTA2-AS1 was closely associated with patients’ lymphatic metastasis status (*P* <  0.001), histological grades (*P* = 0.003), and TNM stage (*P* = 0.005). Specifically, patients with positive lymphatic metastasis status and advanced histological grade and TNM stage showed relatively lower ACTA2-AS1 levels (Table 1). In addition, the significant correlations between miR-532-5p and lymphatic metastasis status (*P* <  0.001), histological grades (*P* = 0.041), and TNM stage (*P* = 0.008) were also revealed (Table 1).

**Table 1:** Association between patients’ clinicopathological features with ACTA2-AS1 or miR-532-5p expression level

	Cases (119)	ACTA2-AS1		*P*	miR-532-5p		*P*
		Low	high		low	high	
Age				0.399			0.762
< 55	56	31	25		26	30	
≥ 55	63	30	33		31	32	
Tumor size				0.561			0.220
< 2	40	22	18		16	24	
≥ 2	79	39	40		41	38	
Lymphatic metastasis				< 0.001			< 0.001
Yes	42	32	10		8	34	
No	77	29	48		49	28	
Histological grade				0.003			0.041
I-II	81	34	47		44	37	
III	38	27	11		13	25	
Family history				0.583			0.725
Yes	44	24	20		22	22	
No	75	37	38		35	40	
Pathological type				0.525			0.655
Ductal invasive	58	28	30		29	29	
Others	61	33	28		28	33	
TNM stage				0.005			0.008
I-II	73	30	43		42	31	
III	46	31	15		15	31	

**Fig. 2:**
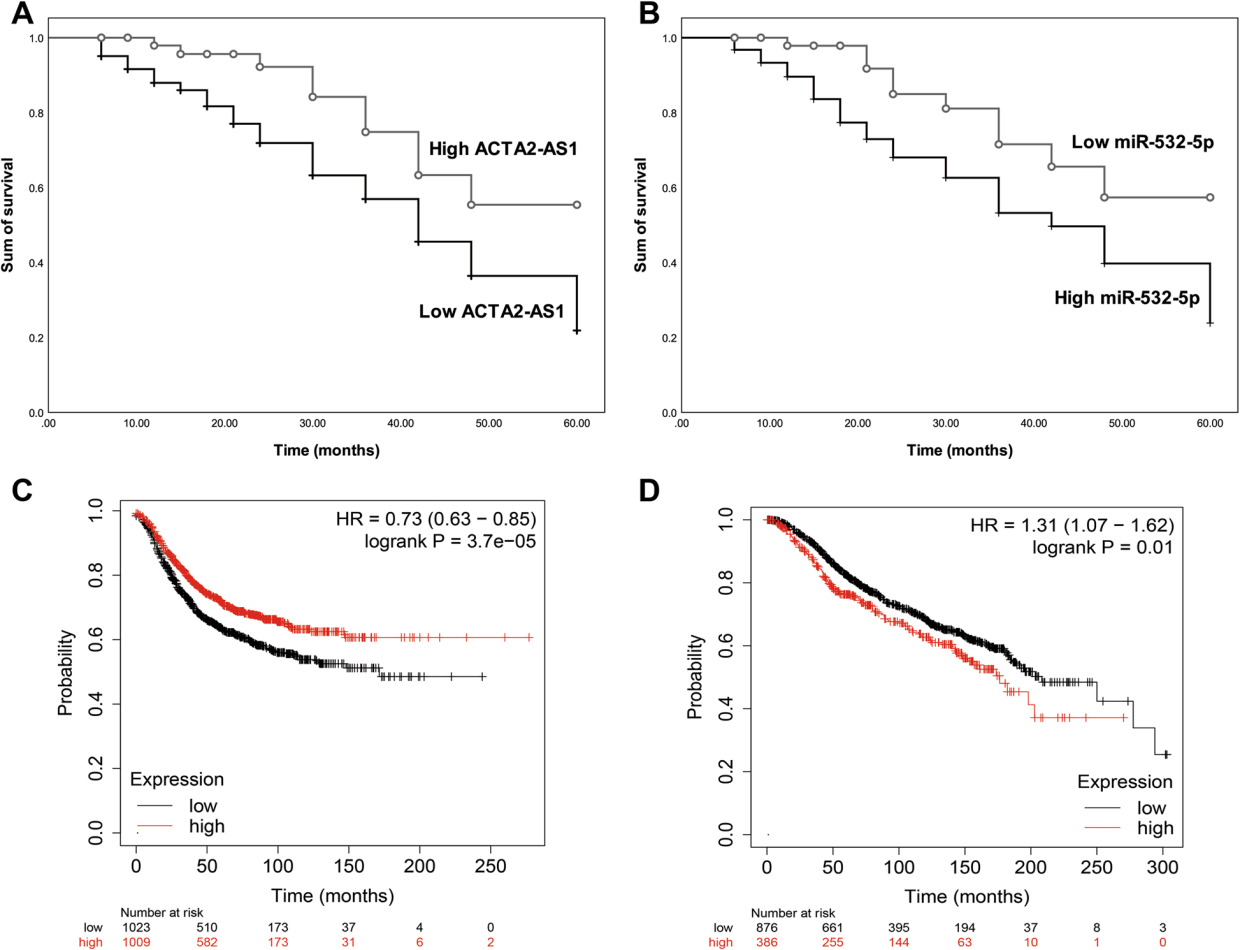
Prognosis of TNBC patients according to the expression of ACTA2-AS1 (**A**) and miR-532-5p (**B**). Both ACTA2-AS1 downregulation (log rank *P* = 0.034) and miR-532-5p upregulation (log rank *P* = 0.018) are associated with the poor prognosis of TNBC patients. The online dataset verified the significant prognostic value of ACTA2-AS1 (**C**) and miR-532-5p (**D**) in breast cancer

According to the obtained outcomes of patients, both the downregulation of ACTA2-AS1 (Fig. 2A, log rank *P* = 0.034) and the upregulation of miR-532-5p (Fig. 2B, log rank *P* = 0.018) were related to the poor prognosis in TNBC patients. Additionally, the significant association of ACTA2-AS1 downregulation (Fig. 2C) and miR-532-5p upregulation (Fig. 2D) with TNBC patients’ prognosis was validated by the online dataset. ACTA2-AS1 (HR = 3.247) and miR-532-5p (HR = 3.214) were demonstrated as two independent prognostic indicators of TNBC as well as the TNM stage (HR = 3.804), histological grade (HR = 3.189), and lymphatic metastasis status (HR = 3.638) according to the results of Cox multivariate regression analysis (Table 2).

**Table 2:** Prognostic value of patients’ clinicopathological features evaluated by Cox regression analysis

	HR factor	95% CI	*P*
ACTA2-AS1	3.247	1.449–7.274	0.004
miR-532-5p	3.214	1.052–9.821	0.005
Age	1.659	0.782–3.520	0.187
Tumor size	1.816	0.726–4.543	0.202
Lymphatic metastasis	3.638	2.182–7.566	0.010
Histology grade	3.189	1.444–7.044	0.040
Family history	1.556	0.690–3.511	0.287
Pathological type	1.838	0.862–3.265	0.101
TNM stage	3.804	1.499–9.650	0.005

### The roles of ACTA2-AS1 and miR-532-5p in the biological function of TNBC cells

**Fig. 3:**
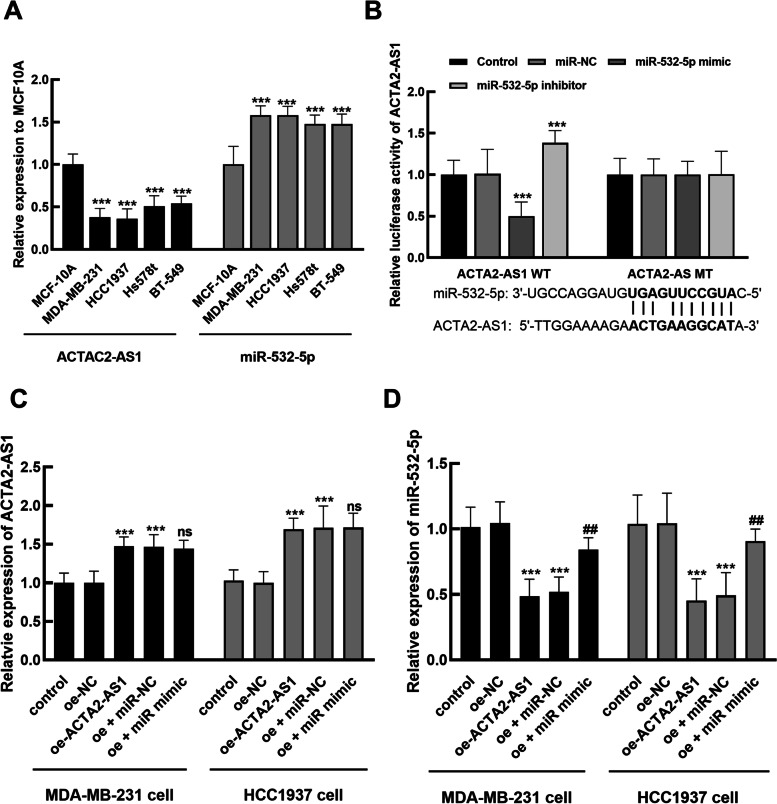
Expression and transfection of ACTA2-AS1 and miR-532-5p in TNBC cells. **A** Significant downregulation of ACTA2-AS1 and upregulation of miR-532-5p were observed in the TNBC cells. ^***^P < 0.001 compared with normal cells. **B** The luciferase activity of WT-ACTA2-AS1 was significantly suppressed by the overexpression of miR-532-5p and enhanced by miR-532-5p silencing. **C**, **D** The transfection of oe-ACTA2-AS1 significantly enhanced ACTA2-AS1 (**C**) and inhibited miR-532-5p (**D**), while the transfection of miR-532-5p mimic reversed the inhibition of miR-532-5p and showed an insignificant effect on ACTA2-AS1. ^***^P < 0.001 compared with control, ^##^*P* < 0.01, ^ns^*P* > 0.05 compared with oe-ACTA2-AS1

Consistent with the abnormal expression levels in TNBC tumor tissues, the reduced ACTA2-AS1 level, and the increased miR-532-5p level were observed in MDA-MB-231, HCC1937, Hs578t, and BT-549 cells compared with MCF-10A (Fig. 3A, *P* < 0.001). The overexpression of miR-532-5p was disclosed to suppress the luciferase activity of WT-ACTA2-AS1, while an opposite effect was found in the knockdown of miR-532-5p on WT-ACTA2-AS1 luciferase activity (Fig. 3B, *P* < 0.001). The MT-ACTA2-AS1 was not influenced by miR-532-5p dysregulation (*P* > 0.05).

The regulatory effect of ACTA2-AS1 on miR-532-5p was evaluated in MDA-MB-231 and HCC1937 cells. It was found that ACTA2-AS1 was dramatically enhanced by the transfection of its overexpression vector (*P* < 0.001), and the transfection of miR-532-5p mimic showed no significant effect on ACTA2-AS1 levels (Fig. 3C, *P* > 0.05). For miR-532-5p, the overexpression of ACTA2-AS1 exerted a significant inhibitory effect on its expression (*P* < 0.001), which was reversed by the transfection of its mimic (Fig. 3D, *P* < 0.01).

**Fig. 4:**
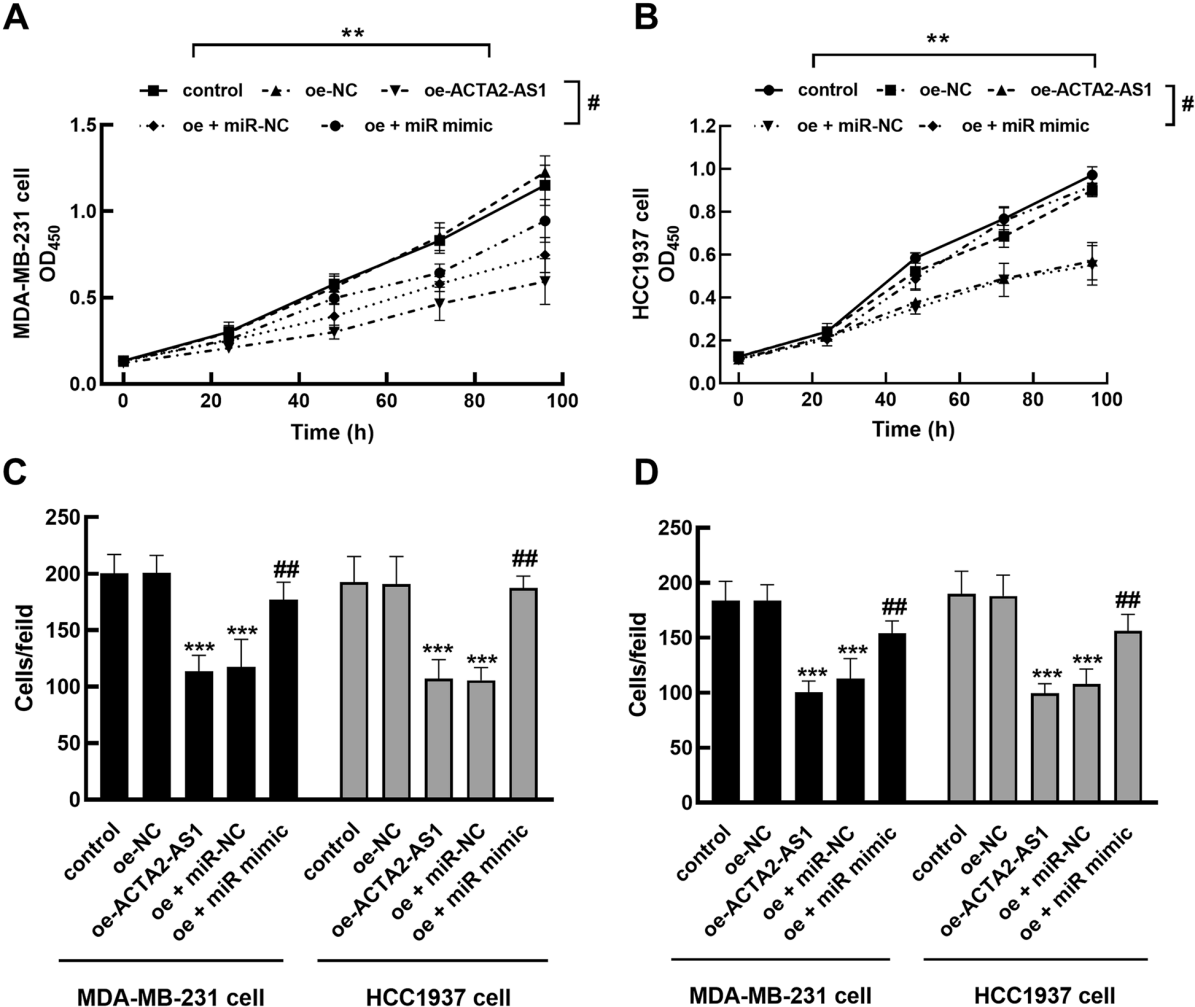
Effect of ACTA2-AS1 and miR-532-5p on cellular processes of TNBC. **A**, **B** ACTA2-AS1 overexpression significantly inhibited the proliferation of MDA-MB-231 (**A**) and HCC1937 cells (**B**), which was alleviated by miR-532-5p upregulation. **C**, **D** ACTA2-AS1 overexpression significantly inhibited the migration (**C**) and invasion (**D**) of MDA-MB-231 and HCC1937 cells, which was reversed by miR-532-5p upregulation. ^**^P < 0.001, ^***^P < 0.001 compared with control, ^#^*P* < 0.05, ^##^*P* < 0.01, compared with oe-ACTA2-AS1

Furthermore, the effects of ACTA2-AS1 and miR-532-5p on the biological function of MDA-MB-231 cells and HCC1937 cells were assessed. Elevated ACTA2-AS1 dramatically suppressed the proliferation of MDA-MB-231 (Fig. 4A) and HCC1937 cells (Fig. 4B, *P* < 0.01), while the overexpression of miR-532-5p could alleviate the inhibitory effect and restore cell viability (*P* < 0.05). Similar to the migration (Fig. 4C and Fig. S1) and invasion (Fig. 4D and Fig. S2) of MDA-MB-231 and HCC1937 cells, ACTA2-AS1 also showed a markedly inhibitory effect (*P* < 0.001), which was reversed by the elevating of miR-532-5p (*P* < 0.01).

## Discussion

Although significant regional and ethnic differences appeared in the incidence of malignant tumors, TNBC still accounts for a higher occurrence than other subtypes of breast cancer [14]. The early screening and progression monitoring are critical factors closely associated with the clinical management of TNBC. Currently, the identification of biomarkers has attracted special attention in tumor research [15]. Increasing evidence confirmed the functional role of abnormally expressed lncRNAs and miRNAs in prognosis prediction and development regulation of TNBC and other malignant tumors [16–19].

Herein, the dysregulation of ACTA2-AS1 and miR-532-5p in TNBC was confirmed. Specifically, the downregulation of ACTA2-AS1 was observed in TNBC, which was negatively correlated with the upregulation of miR-532-5p. Previously, both ACTA2-AS1 and miR-532-5p have been revealed to regulate tumor progression of human cancers and indicate patients’ outcomes. For example, the increased expression of ACTA2-AS1 in cervical cancer was significantly correlated with the advanced FIGO stage of patients, which indicated the malignant development of cervical cancer [20]. In a clinical trait based on the tissues from TCGA, ACTA2-AS1 was also identified as a biomarker of ovarian cancer that is associated with patients’ overall survival [21]. Here, the clinical significance of ACTA2-AS1 in TNBC patients’ illness andprognosis was also leaked out. ACTA2-AS1 showed a close relationship with the risk factors of TNBC development, including patients’ lymphatic metastasis status, pathological grades, and the TNM stage. For miR-532-5p, consistent with its negative correlation with ACTA2-AS1, miR-532-5p was demonstrated as a ceRNA of ACTA2-AS1, which was negatively regulated by ACTA2-AS1. The expression of miR-532-5p was different based on the types of human cancers. In glioma, miR-532-5p was suggested to serve as a tumor suppressor [22]. However, in breast cancer, miR-532-5p was upregulated accelerating the proliferation and migration of breast cancer cells, which is consistent with our obtained results [12]. The high expression of miR-532-5p was also demonstrated to be related to the malignant progression of TNBC patients, behaved as the occurrence of lymphatic metastasis, advanced histological grade and TNM stage. Additionally, miR-532-5p was identified as an indicator of TNBC prognosis together with ACTA2-AS1, which was verified by the online database. Moreover, the insignificant association of ACTA2-AS1 or miR-532-5p with patients’ tumor size and pathological types, which are also critical risk factors of TNBC progression, was observed. This might result from the relatively small sample size and the single center data. Therefore, further multiple center investigations with larger sample size are needed, which would validate the significance of ACTA2-AS1 and miR-532-5p in TNBC and might also provide some new insights.

As the ceRNA of ACTA2-AS1, miR-532-5p was speculated to mediate the function of ACTA2-AS1 like its role during the regulatory effect of other lncRNAs [16, 23]. For instance, LINC01410, as an illustrated sponge of miR-532-5p, promoted the angiogenesis and metastasis of gastric cancer, while miR-532-5p was found to mediate the function of LINC01410 via attenuating the NF-kappaB signaling [24]. In vitro, the overexpression of ACTA2-AS1 was found to restrain the cellular processes of TNBC cells, and miR-532-5p could reverse the inhibitory effect of ACTA2-AS1 overexpression and improve cell viability and metastasis. The interaction of ACTA2-AS1 with miR-532-5p was firstly reported in regulating the development of ovarian cancer, where miR-532-5p mediated the promotor role of ACTA2-AS1 [13]. Consistently, miR-532-5p was also found to mediate the effect of ACTA2-AS1 in TNBC, indicating its involvement during the biological function of ACTA2-AS1 in TNBC cellular processes.

However, the obtained results were based on in vitro cell experiments and clinical tissues, which lack in vivo validation. Establishing corresponding rat models could help further evaluate the function and mechanism of ACTA2-AS1 and miR-532-5p in TNBC development [25–27]. Therefore, further investigation should consider the in vivo validation to complete the role of ACTA2-AS1 and miR-532-5p in TNBC. Additionally, adjuvant therapies might affect the significance of ACTA2-AS1 and miR-532-5p in TNBC onset and development, which was considered an outlook for our future studies.

**Fig. 5:**
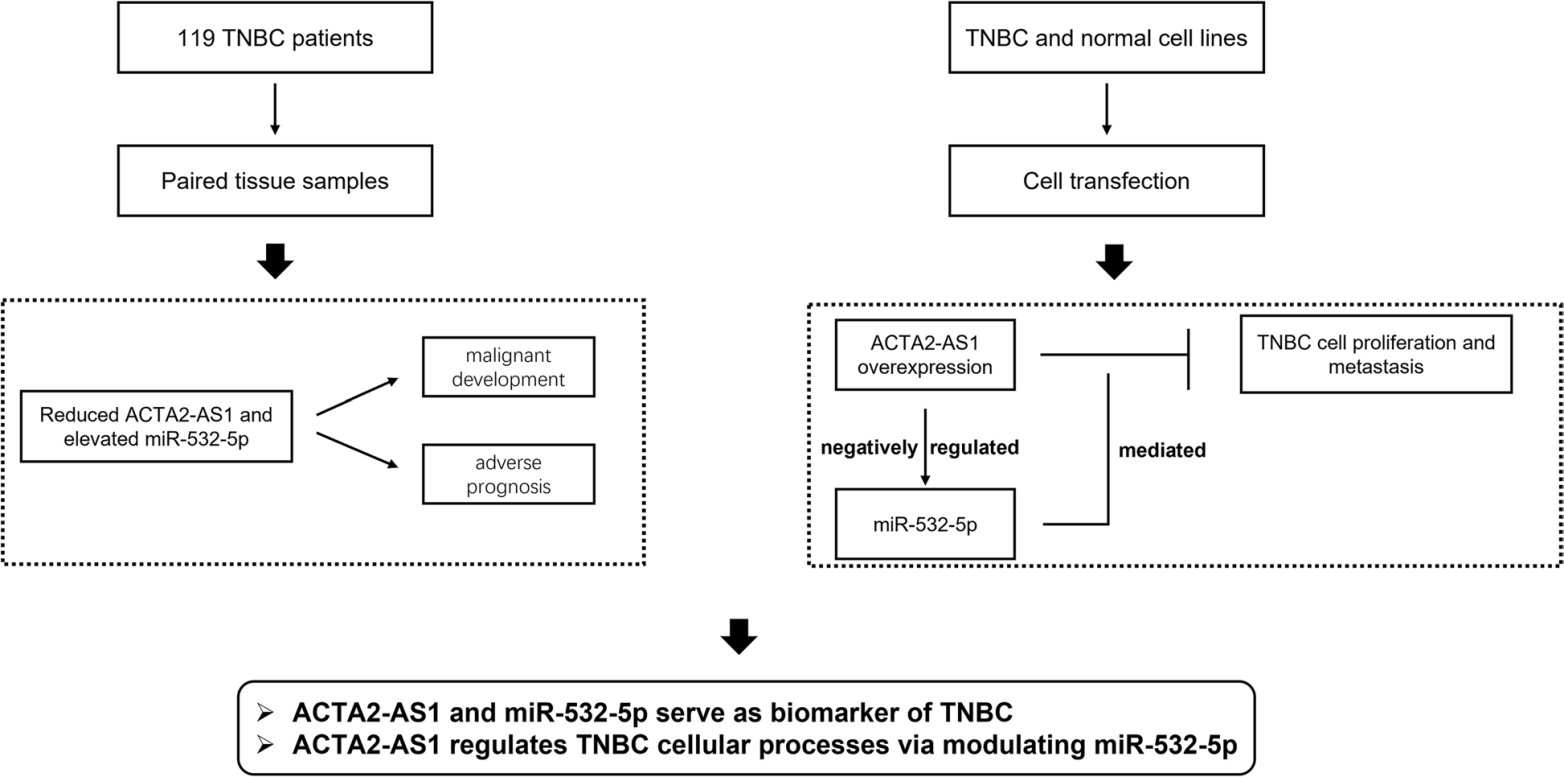
Graphical abstract

According to the above findings, downregulated ACTA2-AS1 and upregulated miR-532-5p in TNBC acted as two biomarkers that indicated malignant tumor progression and poor clinical prognosis. In addition, ACTA2-AS1 served as a tumor suppressor that inhibited the proliferation and metastasis of TNBC cells via sponging miR-532-5p. These results provide two promising biomarkers for the management of TNBC, which could help improve the diagnosis efficiency and progression prediction (Fig. 5).

## Methods

### Study subjects

There were 119 patients with TNBC diagnosed at Fujian Provincial Maternity and Children’s Hospital enrolled in this study from January 2013 to December 2015. The inclusion and exclusion criteria were: 1) patients with negative expression of ER, PR, and HER2; 2) patients were primarily diagnosed with TNBC; 3) patients received surgery for the first time; 4) the clinical data of enrolled patients are completed; 5) patients had received adjuvant therapy before surgery were excluded. The study had been approved by the Ethics Committee of Fujian Provincial Maternity and Children’s Hospital (approval no. 2013188) and had obtained signed informed consent from patients.

The included patients received surgical treatment and the tissue samples (tumor and normal paracancerous tissues) were collected during the surgery. The tissues were confirmed by at least two pathologists (ER, PR < 1% and non-amplified HER-2 on FISH) and stored at − 80 °C. The tumor nuclei and necrosis were assessed with tumor sections.

### Follow-up survey

All patients were followed-up for 5 years after their surgical treatments through outpatient clinical review or telephone. The recurrence, metastasis, and death events were recorded as the end events, and unrelated death was excluded.

### Cell culture

TNBC cell lines (MDA-MB-231, HCC-1937, Hs578t, and BT-20) and a normal cell line MCF-10A were purchased from America Typical culture collection (ATCC, USA) and cultured according to standard protocols. Briefly, the cell culture was carried out in the DMEM medium (catalog# 11995065, Gibco, USA) with 10% FBS (catalog# 10438034, Thermo Fisher Scientific, USA) and 1 × 10^5^ U/L penicillin and streptomycin (catalog# 15070063, Gibco, USA) under sterile conditions at 37 °C with 5% CO_2_.

### Cell transfection

The cultured cells at the logarithmic growth stage with a density of 70–80% confluence were used in the cell transfection. To regulate ACTA2-AS1, cells were transfected with pcDNA 3.1-ACTA2-AS1 (oe-ACTA2-AS1) or corresponding negative controls (oe-NC). pcDNA3.1 and negative controls were obtained from Life Technologies (USA) and the transfected concentration was 50 nM. While miR-532-5p mimic, inhibitor, or negative controls (50 nM) was transfected into cells to regulate miR-532-5p. Cell transfection was conducted with the help of Lipofectamine 2000 (catalog# 11668019, Invitrogen, USA) for 48 h, and then the cells were washed and collected for the following analyses.

### Real-time qPCR

Total RNA was first isolated from cell and tissue samples using TRIzol (catalog#15596018, Invitrogen, USA) according to the manufacturer’s protocols. The purity and concentration of extracted RNA were evaluated by the absorbance at 260 and 280 nm. The reverse transcription into cDNA was performed with 2 μg RNA and the lnRcute lncRNA First-standard cDNA kit (for ACTA2-AS1, catalog# KR202, TIANGEN, China) or the miRcute miRNA First-standard cDNA Synthesis kit (for miR-532-5p, catalog# KR201, TIANGEN, China) according to the manufacturer’s instruments. Then, the PCR amplification was performed with the SYBR Green kit on the Bio-Rad CFX96 real-time PCR detection system (Bio-Rad, Germany) according to the following reaction conditions: 95 °C × 10 min; (95 °C × 30 s, 50 °C × 1 min, 72 °C × 1 min) for 20 cycles; 72 °C × 3 min. The relative expression levels of ACTA2-AS1 and miR-532-3p were calculated with the 2^−ΔΔCt^ method normalized to GAPDH and cel-miR-39, respectively. The sequences of used primers are summarized in Table S1.

### Cell proliferation assessment

Cells (5 × 10^5^ cells/well) were planted in the 96-well plates and maintained in the DMEM culture medium (containing 10% FBS) for a certain period. Then, the cells were washed with PBS and resuspended in the culture medium. CCK8 (catalog# CK04, Dojindo, Japan) was added to each well with a ratio of 1: 10 to the culture medium and incubated for another 2 h. The absorbance at 450 nm was detected with a microplate reader (Molecular Devices, USA).

### Cell metastasis assessment

Cells (5 × 10^5^ cells/well) were seeded into the upper chamber of the Transwell plates (24-well with a pore size of 8 μm) maintained with an FBS-free culture medium at 37 °C with 5% CO_2_ for 48 h and allowed across the chamber plates. The Matrigel (catalog# 356234, corning, USA) was used to coat the upper chambers before the invasion assessment. The bottom chamber was filled with 10% FBS-containing DMEM medium as the chemical attractant. The cells successfully migrating or invading the bottom chamber were stained with 0.1% crystal violet (catalog# C6158, Sigma-Aldrich, USA) and viewed under an optical microscope.

### Dual-luciferase reporter assay

The wild-type and mutant-type ACTA2-AS1 vectors were constructed by cloning with predicted binding sites (on https://starbase.sysu.edu.cn/) or mutant binding sites into the pmirGLO vector (catalog# E133A, Promega, USA). To estimate the interaction between ACTA2-AS1 and miR-532-5p, the constructed vectors were co-transfected with miR-532-5p mimic, inhibitor, or NC into the MDA-MB-231 cell using Lipofectamine 2000 (Invitrogen, USA). The relative luciferase activity of ACTA2-AS1 was analyzed with the Dual-luciferase Reporter System (Promega, USA) and normalized to Renilla.

### Statistical analyses

All data were expressed as mean value ± SD. according to triplicate experiments and determinations. The statistical analyses were carried out with SPSS 26.0 software. The differences between groups were evaluated with a student’s t-test or one-way ANOVA. The clinical data of patients were analyzed with the Chi-square test. The prognostic data proceeded with the Kaplan-Meier and multivariate Cox analysis. Meanwhile, the online Kaplan-Meier plot database (http://www.Kmplot.com) was also employed to further validate the prognostic significance of ACTA2-AS1 and miR-532-5p in breast cancer. The significant differences were represented by *P* < 0.05.

## Supplementary Information

**Additional file 1: Figure S1.** Representative images of the migration of MDA-MB-231 and HCC1937 cells with different transfection treatments.

**Additional file 2: Figure S2.** Representative images of the invasion of MDA-MB-231 and HCC1937 cells with different transfection treatments.

**Additional file 3: Table S1.** The sequences of PCR primers.

**Additional file 4: Table S2.** The list of abbreviations.

## Data Availability

The datasets generated during and/or analyzed during the current study are not publicly available due to the complete project is not yet completed but are available from the corresponding author on reasonable request.

## Abbreviations

TNBC: Triple-negative breast cancer
lncRNAs: Long non-coding RNAs
miRNAs: Short non-coding RNAs
