# Supplementary figures and images for: lncRNA ACTA2-AS1 predicts malignancy and poor prognosis of triple-negative breast cancer and regulates tumor progression via modulating miR-532-5p
Source: BMC Mol Cell Biol. 2022 Jul 27;23:34. doi: 10.1186/s12860-022-00432-7 (PMC9327331; doi:10.1186/s12860-022-00432-7)

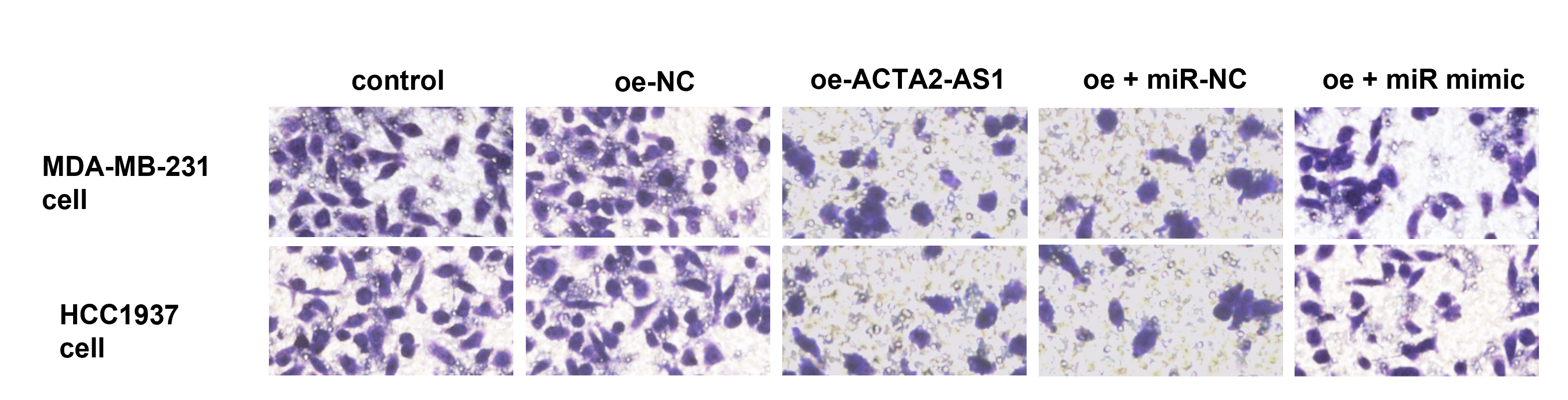

Supplement: Supplementary file 1 — Additional file 1: Figure S1. Representative images of the migration of MDA-MB-231 and HCC1937 cells with different transfection treatments. [file 12860_2022_432_MOESM1_ESM.tif]

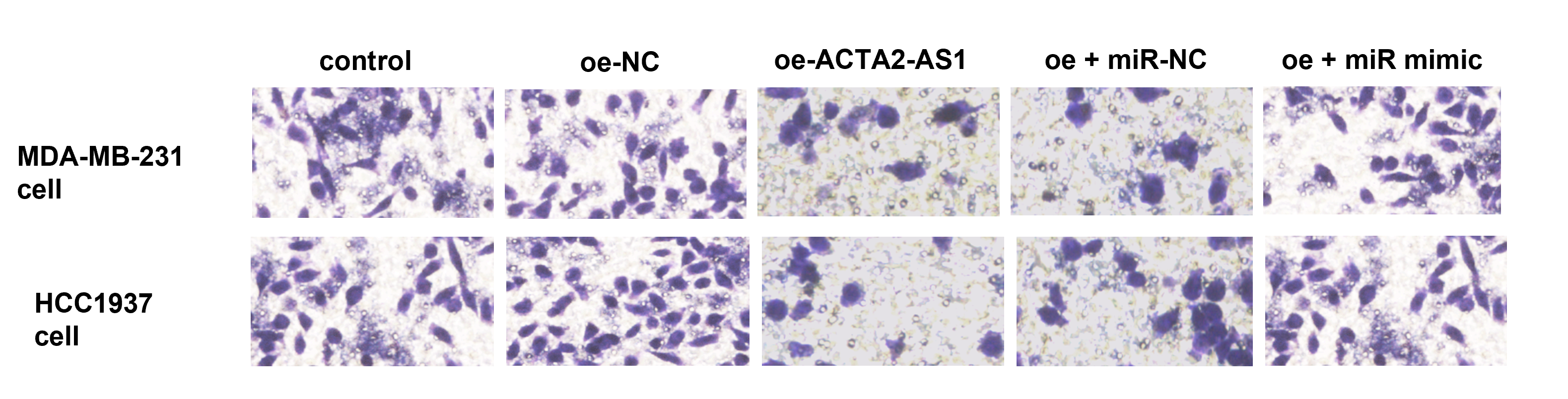

Supplement: Supplementary file 2 — Additional file 2: Figure S2. Representative images of the invasion of MDA-MB-231 and HCC1937 cells with different transfection treatments. [file 12860_2022_432_MOESM2_ESM.tif]
