# Supplementary material for: lncRNA ACTA2-AS1 predicts malignancy and poor prognosis of triple-negative breast cancer and regulates tumor progression via modulating miR-532-5p
Source: BMC Mol Cell Biol. 2022 Jul 27;23:34. doi: 10.1186/s12860-022-00432-7 (PMC9327331; doi:10.1186/s12860-022-00432-7)
Supplement: Supplementary file 3 — Additional file 3: Table S1. The sequences of PCR primers. [file 12860_2022_432_MOESM3_ESM.docx]

| Primers | Sequence |
| --- | --- |
| ACTA2-AS1 Forward | 5’-GTTCTGGAGGCTTGATATGG-3’ |
| ACTA2-AS1 Reverse | 5’-TCCTTCATCGGTAGGCAACAAAC-3’ |
| miR-532-5p Forward | 5’-CTTCCATGCCTTGAGTGTA-3’ |
| miR-532-5p Reverse | 5’-GTGTGGGAGGTAATTAAGATG-3’ |
| GAPDH Forward | 5’-ACCACAGTCCATGCCATCAC-3’ |
| GAPDH Reverse | 5’-TCCACCACCCTGTTGCTGTA-3’ |
| Cel-miR-39 Forward | 5’-UCACCGGGUGUAAAUCAGCUUG-3’ |
| Cel-miR-39 Reverse | 5’-TCACCGGGTGTAAATCAGCTTG-3’ |

Table S1. Sequences of used primers in PCR
