# Supplementary material for: lncRNA ACTA2-AS1 predicts malignancy and poor prognosis of triple-negative breast cancer and regulates tumor progression via modulating miR-532-5p
Source: BMC Mol Cell Biol. 2022 Jul 27;23:34. doi: 10.1186/s12860-022-00432-7 (PMC9327331; doi:10.1186/s12860-022-00432-7)
Supplement: Supplementary file 4 — Additional file 4: Table S2. The list of abbreviations. [file 12860_2022_432_MOESM4_ESM.docx]

| abbreviation | Full name |
| --- | --- |
| LncRNA ACAT2-AS1 | lncRNA actin alpha 2, smooth muscle antisense RNA1 |
| TNBC | Triple-negative breast cancer |
| ER | Estrogen receptor |
| PR | Progesterone receptor |
| HER-2 | Human epidermal growth factor receptor-2 |
| ceRNA | competing endogenous RNA |

Table S2. List of Abbreviations
